# Supplementary material for: Patterns of Intron Gain and Loss in Fungi
Source: PLoS Biol. 2004 Nov 30;2(12):e422. doi: 10.1371/journal.pbio.0020422 (PMC532390; doi:10.1371/journal.pbio.0020422)
Supplement: Table S1 — Also available at http://genes.mit.edu/NielsenEtAl/. (4.3 MB ZIP). [file pbio.0020422.st001.zip › NielsenEtAl/html/490.html]

AN6210.1.NCU08012.1.MG01760.1.FG01919.1


```
 CLUSTAL W (1.82) Multiple Sequence Alignments - Introns Inserted


Sequence 1: NCU08012.1	644 aa
Sequence 2: MG01760.1	630 aa
Sequence 3: FG01919.1	632 aa
Sequence 4: AN6210.1	631 aa
Alignment Length: 655 aa
Number Identitical Residues: 207 aa
Alignment Score (without introns) 12776


MG01760.1 	MAVGLANGRH-AAEEEARAEVDVLNSRLEKTTQLTKKIEACLVRLESTGKSVREVAGPLN
NCU08012.1	MAVGLINGRL-AADEEARAEVDVLNSRLEKTSQLTKKIQACLARLDATGKSVRDVAGPLS
FG01919.1 	MSVGLAGGAHTALDEEARAEVDVLNSRLEKTTQLTKKIQSCLNRLETTGKSVQDVAGPLN
AN6210.1  	----MVAPRDTAFAEES-AEVEVLYANLEKLKRLTKKIQGSLVRLETGGNVVKHAIGPIY
          	    :     :*  **: ***:** :.*** .:*****:..* **:: *: *:.. **: 

MG01760.1 	GETKKLQVLD1I-~-DNVLS------AIE~RLRAPADSKNDEEQIIRMG~PEKADLPNYL
NCU08012.1	GETRKLQVLG~N-~-SKSSGPSSPYLVHQ1KLRSPADSKNDEEQIIRMG~PEKAGLPNYL
FG01919.1 	GETRRLQILG~NN1VDSVLA------AID~RLRQPADSKNDEEQIIRVG~PEKAGLSTYL
AN6210.1  	SNTQSLQITN~NN~IDRVIE------AIE~RLRQPLDAKNREEGVIRAG2PQPNNLPQYL
          	.:*: **: .  .  .          . : :** * *:** ** :** * *:  .*. **

MG01760.1 	NSLKRLNKALVDMKASNLRSNQQTMNDLQRLVTLGTTQLATLFDKLLRSETPRSLEPLHY
NCU08012.1	ASIKRLNKALNDMKASNLRSTQQTVADLQRLVKTGNSQLESSFDKLLRSETPRAIEPLHY
FG01919.1 	ASIKRLGKALAEMQASNLRANQQTMADLSRLIKSGNSQLEHHFETVLRGETPRSVEPLHY
AN6210.1  	AAMRGVNDALMDLTSTNLKSNQKAISEFTSLLGIGNSKLQDLLRLKLG-EHVSPIEPLHY
          	 ::: :..** :: ::**::.*::: ::  *:  *.::*   :   *  *   .:*****

MG01760.1 	ITKD~KPFPLLSRDNVNRLGPIYSFVAGSNRQGKGVGS--ESTIAEVYSEVRGPYLAETL
NCU08012.1	LTKN~MPFPVLSQEKITRLGLMNSYLTGVHQQNTGAGSSQESPVIKIYAEVRAQYLLSTL
FG01919.1 	ITKD~KPFPTLPQDKIARLGLIYSYVVESHHNG-------ASELAHIYAEVRGPYLSASL
AN6210.1  	LTKD1LPFPTIPEDRIAEIAPICAAINSAAVHVPHRGEG-GSPALKIYADVRGPYISTSA
          	:**:  *** :..:.: .:. : : :     :    ...  *   .:*::**. *:  : 

MG01760.1 	ANLAAASVNTAKKKNPD-AVYRAGTNGIGTYAQAMERLFQAEYENITRIFSREDWAPLFQ
NCU08012.1	GNLATASTNTAKKKTPE-AVYKAGTNGMGTYAQAMEGLFLAEYDNICSIFMREDWGPVFQ
FG01919.1 	ANLAAASVNTAKKKSPD-AVYRAGTNGMGTYAQAMEGLFISEYDNVCSVFSREDWGVIFL
AN6210.1  	QNLAIASLNTLKRRVDDESPYKQGTNGIGTYSDALENFIYAEWEAIKRIFTGDHRGLALQ
          	 *** ** ** *::  :.: *: ****:***::*:* :: :*:: :  :*  :. .  : 

MG01760.1 	ATCQNAIVELSRTLREINAHIKVHLNTDCFLAYEIVEIISGMSSRLED----LRAAFAAC
NCU08012.1	ATCQPALVELGRTLRELNSHIKSHITTDCYLAYEIVEIISSLSSNLENRTGELKSSLAAA
FG01919.1 	STCQTALAEQARCLRELNAHIKSHLNTDCYLAYEITEIISALSGKLETRTGELKGALAAA
AN6210.1  	MTCRSAIAEYSKTIRELNDYIRTNLLTDCFLAFEIIDIVTAKSYDIELKTGELKSLFLEA
          	 **: *:.* .: :**:* :*: :: ***:**:** :*::. *  :*  :..*:. :  .

MG01760.1 	LKPVRETAKTSLGELIEDTKRKVANMQSIPADGAPSPVIAETMQRLQTMVEFLRPVSSIM
NCU08012.1	LKPIRETAKSSLVDLLEETKRQVNSLQTLPADGAPTPLATQTMQRLQSMVNFLRPISSIM
FG01919.1 	LKPVRETAKSSLAELLEETRRKVGMLQILPSDGAPIPLVSETMQRLQTMVHFLRPISSIM
AN6210.1  	LRPVRETAKYSLAELLEETKRRAQAIPMLPPNGAPTPLVDKVMRSLIELTGYQKPLASIL
          	*:*:***** ** :*:*:*:*:.  :  :*.:*** *:  :.*: *  :. : :*::**:

MG01760.1 	ISIGNGGWKSLASSRGGG-DALPSLASFDVGANGQEIFADYCSDTIDILLLSLDGKARMM
NCU08012.1	ISIGDGGWKSAAASKGGATDTIPSLVSFDVGADGQEIFAHYCADTIETLLSSLDARARVL
FG01919.1 	ISIGDGGWKANAATNGRSTDAIPSLASFDIGADGKEIFSHYCLDTIEMLLSGLDQKSRVL
AN6210.1  	TSLGDGNWRSTSAS--------SMNTPLDVNPDSDVLFSHFILDVIETLLIALEARARQL
          	 *:*:*.*:: :::        .  ..:*:..:.. :*:.:  *.*: ** .*: ::* :

MG01760.1 	NG-KKPVVGVFIANSIAITERSIRES-DLAPLMET--RLGILETYRKKAKLYYTEPCKDV
NCU08012.1	YQQKKAVIGVFLANNVTVIERMINES-GLVTLLQS--RLQVLDVWRKKATALYTETCKEI
FG01919.1 	MK-SRAVAGVFMANSVVIIGRMVQTS-ELNDLLEN--KLDILEQWRKKATASYTDICKDL
AN6210.1  	HR-TKAAQGVFLSNVFCLVDRAIRSSPELARFLGSPDSVSRIDTFRKRATSTYLDAWKET
          	   .:.. ***::* . :  * :. *. *  :: ... :  :: :**:*.  * :  *: 

MG01760.1 	SMHLFDVIHTSKS---ARPSSGQASADSATILKQLSSKDKESIKNKFTSFNAAFDDMVAR
NCU08012.1	SIHLFDTVHTNRT---ARPGSGQGMVSSASIMKGLSSKDKEKIKGMFTAFNSGFEDMVAR
FG01919.1 	SVHLFDTVHTNRT---NRPTSG--PVDSTSIVKGLGSKDKDKIKEKFTQFNGAFDDMVSR
AN6210.1  	SHYLLDVQYTSHTRGGSRPQSG-GAVDSSAIVKSLSSRDKDAIKDKFKAFNASFDDLVAR
          	* :*:*. :*.:: .. ** ** . ..*::*:* *.*:**: **  *. **..*:*:*:*

MG01760.1 	HKSFSMEREVRQMFARDMQQMLEPLYVRFWDRYHEVDKGKGKYVKYDKAAIAAVFASLY-
NCU08012.1	HKQFTMEKEVRQMLAQDVQHMLEPLYNRFWDRYHEIDKGKGKYVKYDKGSIAAVFRSLY-
FG01919.1 	HKSYSMEREVRRIFGEDIRQKLQPLYERFWDRYHEIDKGKGKYVKYDKTSIAAVFASLAS
AN6210.1  	HKSFYMEREVRSVLAREVQAVLEPLYARFYDRYHELDKGRGKYTKYDKGSLSAQLASLQ-
          	**.: **:*** ::..:::  *:*** **:*****:***:***.**** :::* : **  

NCU08012.1	
MG01760.1 	
FG01919.1 	
AN6210.1  	
          	
```
